# Supplementary figures and images for: SH-SDS: a new static-dynamic strategy for substation host security detection
Source: PeerJ Comput Sci. 2024 Nov 22;10:e2512. doi: 10.7717/peerj-cs.2512 (PMC11623280; doi:10.7717/peerj-cs.2512)

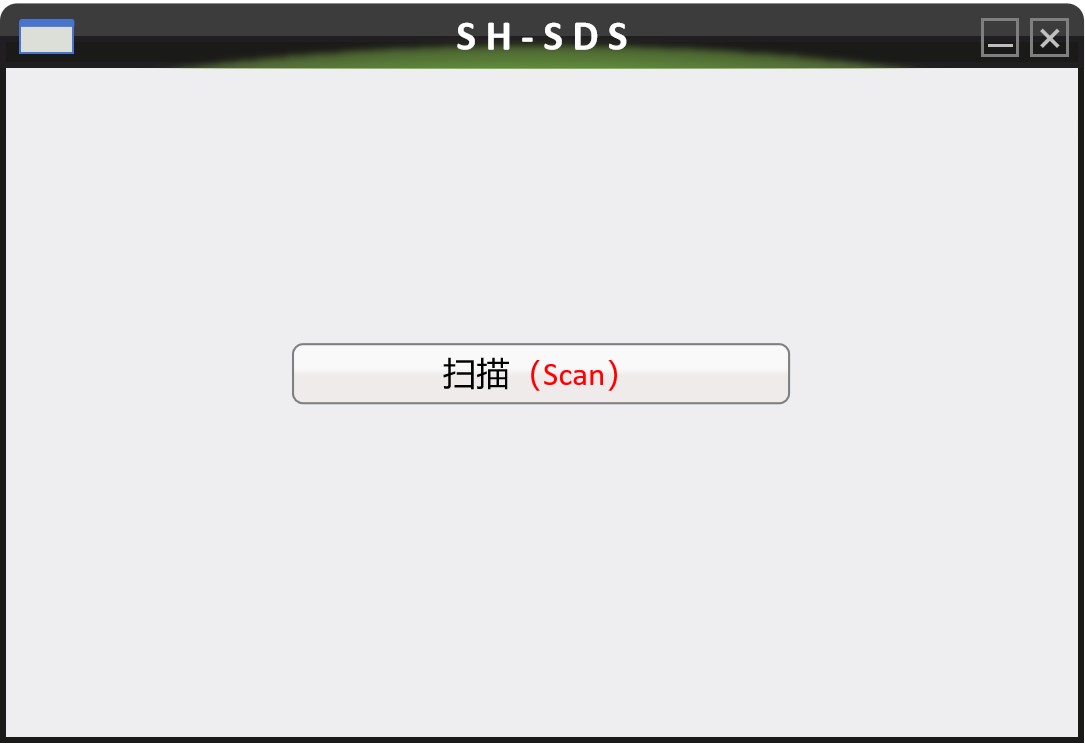

Supplement: Supplemental Information 3 [file peerj-cs-10-2512-s003.zip › SH-SDS-main/UI/main.jpg]

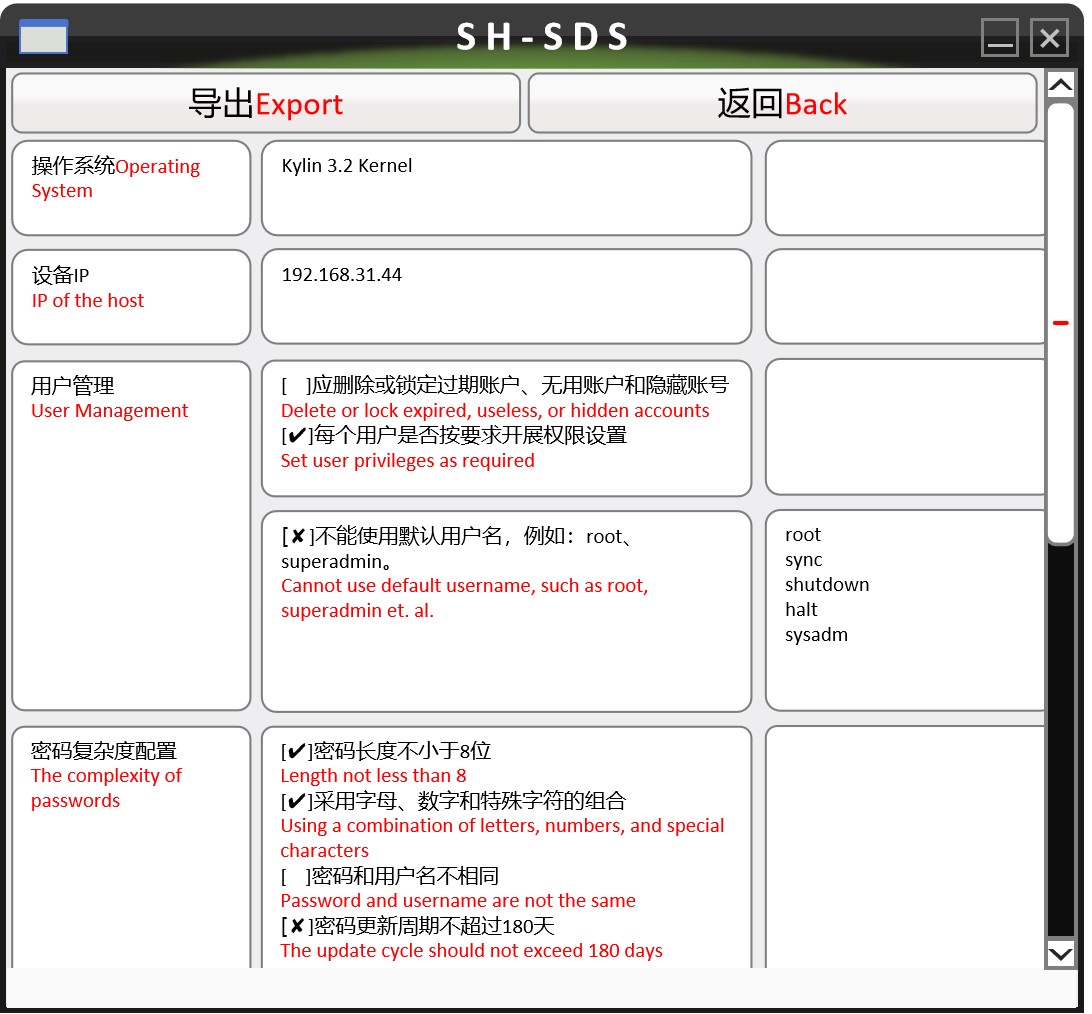

Supplement: Supplemental Information 3 [file peerj-cs-10-2512-s003.zip › SH-SDS-main/UI/operation.jpg]
